# Hsa-miR-30a-3p attenuates gastric adenocarcinoma proliferation and metastasis via APBB2

**DOI:** 10.18632/aging.203197

**Published:** 2021-06-28

**Authors:** Kun Zhou, Dachun Cao, Yu Wang, Lei Wang, Xiangjun Meng

**Affiliations:** 1 Department of Gastroenterology, Ninth People’s Hospital, College of Stomatology, Shanghai Jiao Tong University School of Medicine, Shanghai 200011, China

**Keywords:** gastric adenocarcinoma, APBB2, hsa-mir-30a-3p, proliferation

## Abstract

Background: There is a well-established relationship between cell cycle progression and the development of stomach adenocarcinoma. This study aimed to elucidate the molecular mechanism and biological function of APBB2 in gastric cancer.

Methods: Gastric adenocarcinoma (GA) data were downloaded from the TCGA-GA and GEO databases and analyzed to explore differentially expressed miRNAs and mRNAs. Moreover, potential target mRNAs were also predicted. The relative level of gene and protein expression in GA cell lines and gastric mucosa cells was detected by q-PCR and Western blot, respectively. Moreover, the influence of APBB2 on proliferation, metastasis, and cell cycle changes in SGC-7901 and BGC-823 cells was evaluated. The binding relationship between the target miRNA and mRNA was confirmed with a dual-luciferase reporter assay.

Results: High APBB2 expression was detected in GA patients, indicating that it may be represent a predictive biomarker for poor prognosis. Related experiments confirmed that APBB2 silencing inhibited GA cellular functions, including proliferation, cell cycle progression, migration, and invasion. In addition, to explore the molecular mechanism, our results indicated that the binding sites were located at hsa-mir-30a and the 3′-UTR of APBB2, suggesting that hsa-mir-30a can regulate the expression of APBB2. The biological functions of hsa-mir-30a were also evaluated. Hsa-mir-30a overexpression attenuated the proliferation and metastasis of cancer cells. In rescue experiments, hsa-mir-30a was confirmed to reverse the cell cycle promoting function associated with APBB2 overexpression.

Conclusion: Our findings show that hsa-mir-30a can attenuate the development of GA by down-regulating APBB2 expression.

## INTRODUCTION

Gastric adenocarcinoma (GA) is a subtype of stomach adenocarcinoma (STAD) that represents a significant cause of cancer-related mortality. [1–3] Despite the overall incidence of GA, retrospective studies have confirmed an increasing incidence of GA among young people aged between 35 and 44 years. [4] Furthermore, despite developments in treatment strategies and early detection methods, the prognosis of GA patients remains poor [5], with a 5-year survival rate of only 29% [5]. Therefore, there is an urgent need to explore novel methods of achieving accurate early stage detection and develop effective treatments for GA.

Cell cycle progression plays a key role in cell proliferation and represents an essential feature of cancer development and progression. In bladder cancer, APBB2 plays a dual regulatory role, mediating the cell cycle via the CDK6 and MET pathways [6]. APBB2 (FE65L1) has been well-characterized for its function in amyloid precursor protein processing [7] and was also recently confirmed to play a role in the cell cycle [8]. Moreover, related research has also demonstrated that APBB2 overexpression in PC12 cells can induce cell cycle progression by inhibiting thymidilate synthase (TYMS, NC_000018.9) [8]. Moreover, Peille et al. [9] conducted an analysis of the Gene Expression Omnibus (GEO) database, and found that the expression of several genes, including APBB2, may influence the prognosis of GA patients. Despite these advances made in previous research, the specific molecular mechanism by which APBB2 regulates the development of GA remains poorly understood.

The results of this study showed that miR-30a-mediated APBB2 expression was essential for STAD progression. In addition, APBB2 up-regulation was associated with cell cycle progression in STAD cells, whereas APBB2 silencing attenuated GA cell proliferation. Therefore, exploring the molecular mechanism of APBB2 may provide a novel anti-tumor treatment strategy for GA.

## MATERIALS AND METHODS

### Bioinformatics

GSE112264 (Normal: 41, Tumor: 809) datasets were obtained from the GEO database (https://www.ncbi.nlm.nih.gov/geo/). The SVA package was used for batch correction. To obtain the differentially expressed miRNAs, the “edgeR” package (|logFC| > 2.5, padj < 0.05) was used to compare the differences between the control group and tumor group. The mRNA profiles (Normal: 34, Tumor: 415) were obtained from the TCGA-GA clinical database (https://portal.gdc.cancer.gov/). The “edgeR” package (|logFC| > 1, padj < 0.05) was used to analyze the differentially expressed miRNAs (DEmiRNAs) between the normal group and tumor group. The potential upstream miRNAs of APBB2 were predicted according to the mirDIP and starBase databases. DEmiRNAs were used to explore the binding sites between the miRNAs and target mRNA. A Kaplan-Meier analysis was used to calculate the overall survival (OS) probability associated with the target mRNA and corresponding miRNAs based on the TCGA-GA dataset.

### Cell lines and culture

Human GA cell lines (SGC7901, MGC803, and MGC823) and a human gastric mucosa cell line (GES-1) were purchased from Bluefcell (Bluefcell, Shanghai, China). Complete medium consisting of Dulbecco’s Modified Eagle Medium (DMEM; Sigma, St. Louis, MO, USA) containing 10% fetal bovine serum (FBS; Gibco, Carlsbad, CA, USA) was used for cell culture. All cell lines were cultured in an incubator containing 5% CO_2_ at 37°C. After recovery, cells at passages 2–4 were used in experiments. All laboratory procedures were approved by the ethical committee of Ninth People’s Hospital, Shanghai Jiao Tong University School of Medicine.

### Cell transfection

Mimic NC, miR-30a-3p mimic, sh-NC, sh-APBB2 (sh-APBB2-1, sh-APBB2-2, and sh-APBB2-3), over-expressed (OE) plasmid of APBB2 (OE-APBB2) and OE-NC plasmid were obtained from Genomeditech (Shanghai, China) and transfected into GA cell lines using Lipofectamine 3000 (Thermo Fisher Scientific, Inc.) in accordance with the manufacturer’s instructions.

### RNA extraction and qRT-PCR

Total RNA was obtained using an Axygen RNA Miniprep Kit (Axygen, USA) in accordance with the manufacturers’ instructions. A NanoDrop 2000/2000C spectrophotometer was used to test the RNA purity and concentration at wavelengths of 260 nm or 280 nm. A PrimeScript™ RT Reagent Kit (TaKaRa Biotechnology) was used to reverse transcribe 2 μg of the extracted RNA into cDNA. The resultant cDNA was used as a template in the TB Green^®^ Premix Ex TaqTM Kit (TaKaRa Biotechnology) master mix and qPCR reactions were performed on a StepOnePlusTM Real-Time PCR System. The following human primer sets were used: human APBB2: forward, 5-ATGGGACTGCGGAAGAGAAA-3 and reverse, 5-GCCCCTGTTTTCGGATGATC-3; human GAPDH: forward, 5-CCAGAACATCATCCCTGCCT-3 and reverse, 5-CCTGCTTCACCACCTTCTTG-3.

### Colony formation assay

A colony formation assay was used to estimate the level of cellular proliferation. At 48 h post-transfection, 1 × 10^3^ cells/well were seeded into six-well plates and incubated at 37°C for approximately 9 days. The cell colonies were fixed in 5% paraformaldehyde for 10 min and the cell colonies were stained with 0.5% crystal violet. The number of colonies was calculated using ImageJ software.

### Flow cytometry analysis

Adherent cells were collected using trypsin and washed twice in PBS. The cells were stained with propidiumiodide for analysis via flow cytometry (PI; Sigma, St Louis, MO, USA). The proportion of cells at different time points were calculated by FCM (Beckman Coulter, Brea, CA, USA).

### Transwell assay

The migration and invasion ability of GA cells was evaluated by transwell experiments using transwell chambers (Corning Inc., Corning, NY, USA). A total of 1 × 10^5^ cells were seeded into the upper chamber with DMEM. Complete medium was placed into the lower chambers. After a 10 h incubation, the cells in the upper surface of the upper chambers were wiped off. The upper chambers were stained by crystal violet to observe the migrated cells.

### Dual-luciferase reporter gene assay

Luciferase reporter vectors based on psicheck2 were constructed including wild-type APBB2 (APBB2-WT) and mutant-type APBB2 (APBB2-MUT) at the miR-30a-3p binding sites. HEK293T cells were seeded into 24-well plates and co-transfected with the APBB2-WT/APBB2-MUT vector, miR-30a-3p mimic, or NC mimic via Lipofectamine 2000. After transfection for 48 h, the cell lysates were collected and luciferase activity was measured by using a dual-luciferase reporter gene assay system (Promega).

### Statistical analysis

GraphPad Prism 6.0 (Graphpad Software Inc.) was used for all statistical analyses. All values are presented as the mean ± standard deviation (SD). Differences between the experimental and control groups were evaluated using a Student's *t* test. The results for multiple group comparisons were analyzed using a Scheffe's test and one-way analysis of variance (ANOVA) with SPSS 22.0 software (SPSS Inc., USA). A Pearson χ^2^ test was used to analyze the relationship between miR-30a-3p and APBB2 expression. Values were determined to be significant at ^*^P < 0.05, ^**^P < 0.01, and ^***^P < 0.001.

## RESULTS

### High APBB2 expression in GA patients is associated with a poor prognosis

**Figure 1:**
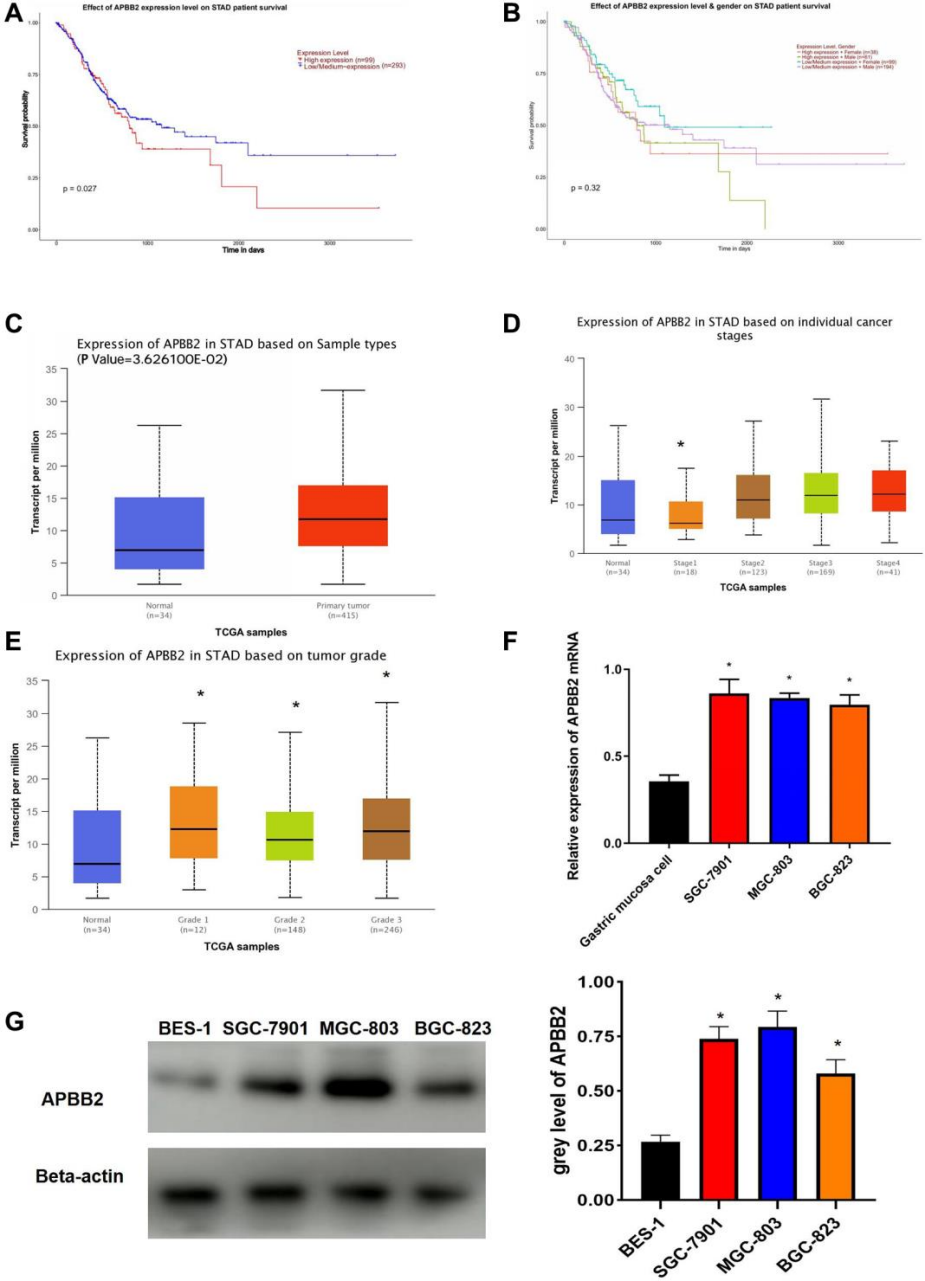
**APBB2 expression and associated prognosis in GA.** (**A**) Survival curves of APBB2 expression to evaluate patient prognosis in the TCGA-GA dataset. Red represents the high expression group and blue indicates low expression. (**B**) Survival curves of APBB2 expression to evaluate male and female patient prognosis in the TCGA-GA dataset. (**C**) Compared with the normal group (blue), high levels of APBB2 expression was observed in the tumor samples (red). (**D** and **E**) Box plots of APBB2 expression at different clinical stages, N stages of GA. (**F** and **G**) The level of APBB2 mRNA and protein expression in BES-1 and GA cell lines (SGC-7901, MGC-803, and BGC-823). ^*^P < 0.05.

Kaplan-Meier analysis indicated that high APBB2 expression indicated poor OS (Figure 1A). The sex-specific survival analysis for APBB2 shown poor OS in both male and female patients with high-expressed APBB2 (Figure 1B). Meanwhile, those difference show no statistical significance. Moreover, an evaluation of the TGCA-GA database confirmed that APBB2 expression was higher in the tumor tissue compared to normal tissue (Figure 1C). An analysis of the patients’ clinical information revealed that APBB2 expression was increased in conjunction with the progression of clinical stages (Figure 1D) and T stages (Figure 1E) of GA. In accordance with the TGCA database analysis, the Western blot results showed that the GA cell lines had higher levels of APBB2 protein expression than that of the BES-1 cell line (Figure 1F and 1G).

### Down-regulation of APBB2 attenuates the biological function of GA cells

**Figure 2:**
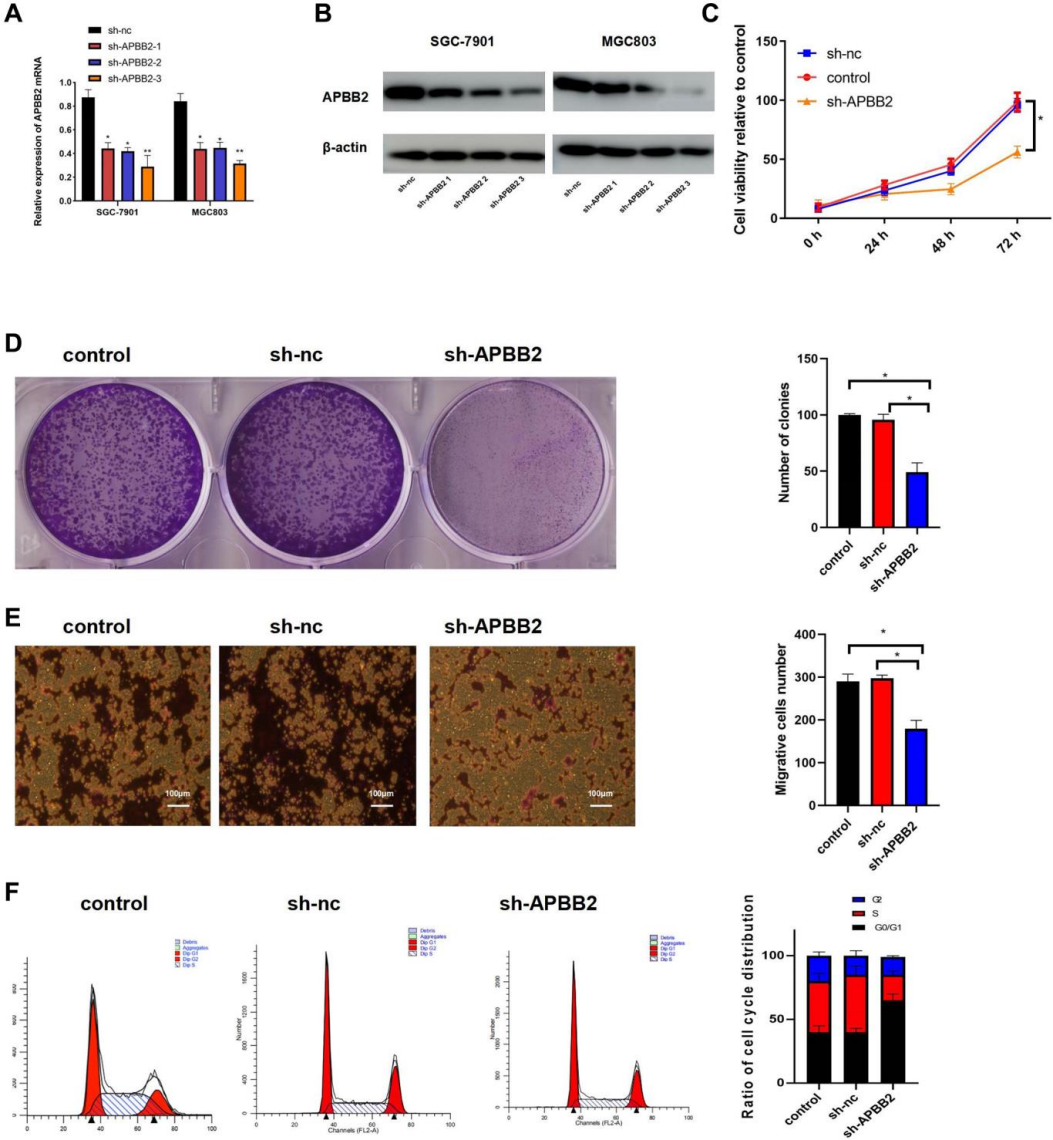
**The down-regulation of APBB2 attenuates proliferation, invasion, and cell cycle progression in GA cell lines.** (**A** and **B**) evaluation of APBB2 expression by PCR and Western blot in GA cell lines following treatment with APBB2-sh-RNA 1,2,3. (**C**) Cell viability was evaluated using an MTT assay. (**D**) GA cell proliferation was determined by a colony formation assay. (**E**) A transwell assay was used to determine the invasion ability of GA cells (4×). (**F**) FCM was used to analyze the proportion of GA cells in each phase of the cell cycle (APBB2-silenced cells and NC cells). ^*^P < 0.05.

The level of APBB2 mRNA expression was down-regulated in SGC-7901 and MGC803 cells using sh-APBB2 transfection (Figure 2A). The Western blot images showed similar results at the protein level (Figure 2B). Since sh-APBB2-3 was associated with the most significant decrease in SGC-7901 and MGC-803, it was selected for subsequent experiments. The down-regulation of APBB2 expression was found to attenuate the proliferation (Figure 2C), colony formation (Figure 2D), and invasion ability (Figure 2E) of SGC-7901 cells. Compared with the control group, there was a significant increase in the proportion of cells in the G0/G1 phase of the cell cycle in the sh-APBB2 group as assessed by FCM (Figure 2F). These findings indicate that the inhibition of GA cell proliferation was induced by decreased APBB2 expression.

### APBB2 is a target gene of miR-30a-3p

**Figure 3:**
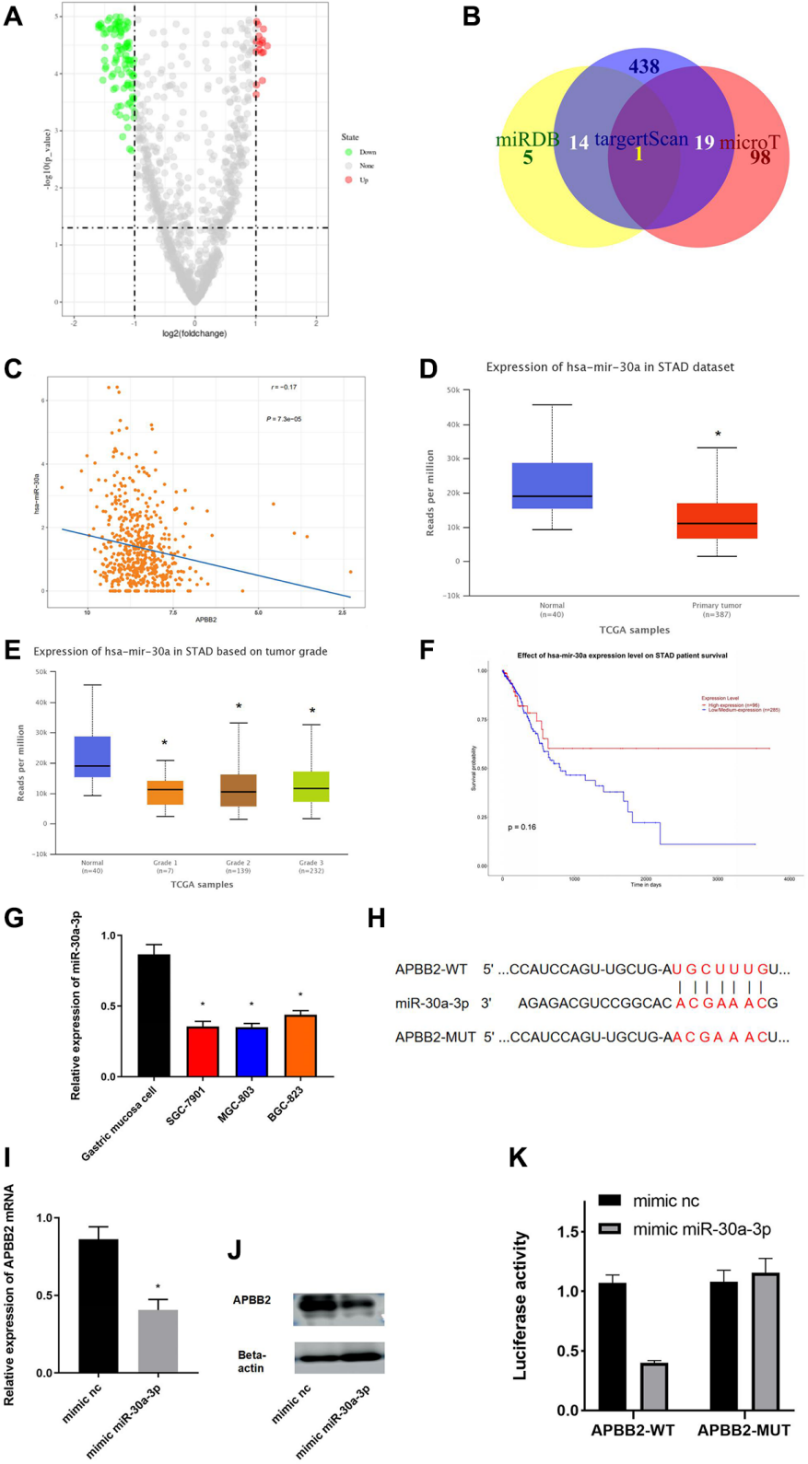
**miR-30a-3p down-regulates APBB2.** (**A**) DEmiRNA volcano map of the normal group and tumor group based on the GEO dataset. (**B**) Venn diagram of predicted down-regulated DEmiRNAs upstream of APBB2. (**C**) Pearson correlation analysis of APBB2 and miR-30a-3p. (**D**) The level of miR-30a-3p expression was down-regulated in the tumor group (red) compared with the normal samples (blue). (**E**) Box plots of miR-30a-3p expression associated with the different clinical stages of GA. (**F**) Survival curves associated with miR-30a-3p expression as an indicator of prognosis. Red indicates the high expression group and blue indicates the low expression group. (**G**) Expression of miR-30a-3p in BES-1 and GA cell lines (SGC-7901, MGC-803, and BGC-823). (**H**) Binding sites of miR-30a-3p and the 3′UTR of APBB2. (**I** and **J**) Results of the mRNA and protein levels were used to evaluate the effect of miR-30a-3p on APBB2. (**K**) A dual-luciferase reporter gene assay was used to determine the targeted binding of miR-30a-3p and APBB2. ^*^P < 0.05.

To explore the mechanism of APBB2 expression in GA, an in-depth analysis of the regulatory pathways located upstream of APBB2 was conducted. A total of 89 different miRNAs were obtained to perform a differential analysis (Figure 3A). The potential upstream miRNAs of APBB2 were predicted using miRDB, targetscan, and microT databases. miR-30a-3p was obtained from the inter-section of 66 down-regulated miRNAs (Figure 3B). A Pearson correlation analysis revealed that there was a negative correlation between miR-30a-3p and APBB2 (Figure 3C). An analysis of the TCGA-GA database revealed low miR-30a-3p expression in the tumor tissues (Figure 3D). In addition, miR-30a-3p expression was decreased in relation to the progression of clinical stages (Figure 3E) of GA. The above evidence was in accordance with the data gained from other cell lines (Figure 3E). A Kaplan-Meier analysis of TCGA-GA dataset revealed that GA patients exhibiting higher miR-30a-3p expression were associated with a longer survival period (Figure 3F).

The binding regions between miR-30a-3p and APBB2 were explored using miRDB and starBase (Figure 3G). APBB2 protein and mRNA expression confirmed that miR-30a-3p played a regulatory function in SGC-7901 cells (Figure 3H). APBB2 expression was significantly down-regulated following treatment with the miR-30a-3p mimic (Figure 3I and 3J). The results of the dual-luciferase reporter gene assay showed that the miR-30a-3p mimic could effectively down-regulate luciferase activity in APBB2-WT cells (Figure 3K). These results confirmed that APBB2 gene expression was down-regulated by miR-30a-3p in GA.

### APBB2 overexpression can reverse miR-30a-3p-mediated attenuation of GA cell functionality

**Figure 4:**
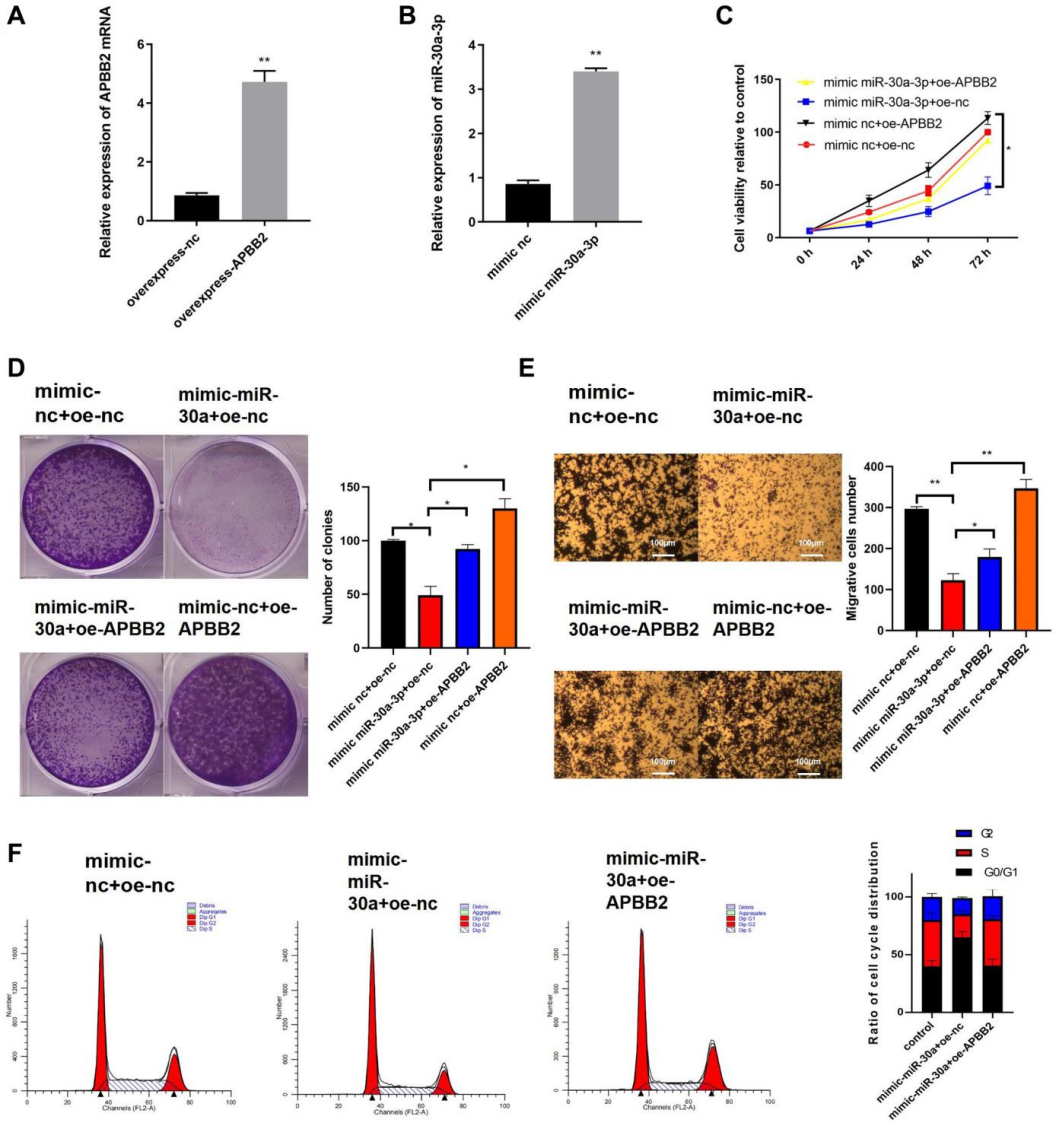
**The overexpression of miR-30a-3p inhibits GA development, which can be reversed by APBB2.** (**A**) Expression of miR-30a-3p and APBB2 in transfected cells was detected by qRT-PCR. (**B**) An MTT assay was used to determine cell viability at 24 h, 48 h, and 72 h, respectively. (**C**) The proliferation of GA cells was determined by a colony formation assay. (**D** and **E**) A transwell assay was used to determine the migration and invasion ability of GA cells(4×); (**F**) The proportion of GA cells in each stage of the cell cycle was analyzed by FCM. ^*^P < 0.05; ^**^P < 0.01.

Next, we further explored the effects of miR-30a-3p expression on APBB2-related cell functions. The efficiency of the APBB2 overexpression (Figure 4A) and miR-30a-3p mimic (Figure 4B) was evaluated. Compared with the control group, the cell proliferation (Figure 4C), colony formation (Figure 4D), and transwell (Figure 4E) assays showed that APBB2 overexpression promoted GA cell proliferation, cell viability, and invasion, which could be inhibited by the miR-30a-3p mimic. Moreover, the cell cycle FCM results also confirmed that the miR-30a-3p mimic effectively blocked GA cells in the G0/G1 phase, which could attenuate GA cell proliferation (Figure 4F). Together, our results demonstrate that miR-30a-3p inhibited the biological function of GA cells, which could be reversed by APBB2 overexpression.

## DISCUSSION

Our results reveal that APBB2 is up-regulated in GA and promotes the biological function (i.e., metastasis and proliferation) of GA cells. Further investigation into the associated mechanism demonstrated that miR-30a-3p regulates APBB2 expression. Traditionally, APBB2 has been regarded as an Alzheimer disease protein associated with cognitive impairment [10]. Recent studies have reported that APBB2 is also overexpressed in several tumors, including breast cancer [11]. In our study, the results of the TCGA-GA mRNA dataset analysis showed significant differences in APBB2 expression between normal and tumor tissues. The RT-PCR and Western blot results in GA cell lines were in line with the findings from the TCGA-GA database analysis. To confirm the essential role of APBB2 in GA, a Kaplan-Meier survival analysis based on the TCGA-GA dataset revealed a negative relationship between APBB2 and patient survival. Moreover, APBB2 expression was increased alongside the progression of clinical stages, N stages and T stages. These results suggest that APBB2 may represent a predictive biomarker of survival and facilitate an early diagnosis of patients with GA. Moreover, APBB2 shRNA was used to explore the function of APBB2 in GA, and was found to significantly inhibit the proliferation, migration, and invasion abilities of the GA cells. The proportion of cells in the G0/G1 phase was significantly increased following treatment with APBB2 shRNA.

It has been found that miRNA plays an essential role in multiple biological functions, including cellular migration, proliferation, autophagy, and transformation [12]. miRNA functions by suppressing mRNA translation through binding to the 3′UTR structure of the target gene mRNA [13]. A bioinformatics analysis of miRNAs in the GEO database was used to explore the potential upstream regulatory mechanism of APBB2. Our results showed that miR-30a-3p had targeted binding sites with APBB2 and was expressed at relatively low levels in GA. Our subsequent experiments demonstrated that miR-30a-3p could suppress APBB2 expression in GA cell lines, indicating that it can attenuate GA development as a tumor suppressor.

In addition, miR-30a-3p has been reported to play a key role in several types of cancer [14–16]. In gastric cancer, miR-30a-3p mediates cancer cell invasion via STAT3/MMP11 signaling [17]. In liver cancer cells, miR-30a-3p attenuates proliferation via targeting DNMT3a [18]. Moreover, miR-30a-3p was found to down-regulate ATG 12-mediated invasion and proliferation in renal cancer cells [19]. In this study, we first confirmed whether miR-30a-3p can regulate APBB2 at the cellular level in GA cells. Our results demonstrate that the biological functions (e.g., proliferation) of GA cells were significantly inhibited by miR-30a-3p overexpression. Furthermore, this inhibition induced by miR-30a-3p could be reversed by plasmid-induced APBB2 overexpression. Since miR-30a-3p is a tumor-derived exosomal miRNA, it may represent a potential biomarker that can be used as an early stage diagnostic for non-small cell lung cancer [20]. Thus, future research should explore the potential possibility of targeting the miR-30a-3p/APBB2 axis as a means of improving the prognosis of GA patients.

APBB2 plays an important role in cancer cell proliferation. Our findings reveal that mir-30a-3p can regulate the malignant process of GA via suppressing APBB2-mediated cell cycle progression. Accordingly, this research confirmed that APBB2 may be used as a novel biomarker for the early diagnosis and therapeutic targeting of GA.

### Availability of data and materials

The datasets used and/or analysed during the current study available from the corresponding author on reasonable request.

### Ethics approval and consent to participate

The study design was approved by the Ethical Committee of Ninth People’s Hospital, Shanghai Jiao Tong University School of Medicine.
